# Acceleration, Deceleration and Dynamic Stress Load in Elite Hurling: A Between-Quarter and Between-Position Comparison

**DOI:** 10.3390/sports9010010

**Published:** 2021-01-12

**Authors:** Damien Young, Giuseppe Coratella

**Affiliations:** 1 Limerick Institute of Technology, Thurles Campus, E41 PC92 Thurles, Tipperary, Ireland; damien.young@hotmail.com; 2 Department of Biomedical Sciences for Health, Università degli Studi di Milano, 20133 Milano, Italy

**Keywords:** match-play running performance, team sport, GPS, elite players, positions

## Abstract

This study described the decrement in accelerations, decelerations and dynamic stress load (DSL) between quarters in elite hurling. GPS (10-Hz) were used to record data from 42 players over 22 games (2018–2020 season). The number of accelerations and decelerations and DSL between quarters were assessed. Accelerations and decelerations were greater in Q1 than Q2 (ES = 0.28 and ES = 0.44, respectively), and Q4 (ES = 0.57 and ES = 0.60, respectively), and in Q3 compared to Q4 (ES = 0.50 and ES = 0.44, respectively). The DSL was 56 ± 21 AU in Q1, 56 ± 20 AU in Q2, 52 ± 20 AU in Q3 and 56 ± 24 AU in Q4. There was a decrease in DSL in Q3 compared to Q1 (ES = −0.20) and Q2 (ES = −0.20). Each position experienced a temporal decrease in at least one quarter (ES = 0.43–1.46) in all metrics except full-backs’, half-backs’ and full forwards’ accelerations, midfielders’ decelerations and midfielders’ and half forwards’ DSL. Current data show temporal decrements in running performance in Q2 and Q4 and DSL in Q3. Players should be conditioned to minimize the drop-off in running performances following the third quarter.

## 1. Introduction

Hurling is an Irish stick and ball field-sport (playing area: 140 m × 90 m) played over a 70-minutes duration, divided between two 35-minute halves. Each team (*n* = 2) of 15 players (1 goalkeeper and 14 outfield players) aim to outscore the opposing team by striking the ball through a set of goalposts [1]. The team receives one point and three points for successfully striking the ball over or under the crossbar, respectively. The ball can be struck (~80 m) from one end of the field to the other very quickly. Frequently, this results in an intense contest for the ball, where players must accelerate and decelerate to gain possession. The players are divided into five positional lines, full backs, half backs, midfielders, half forwards, and full forwards [2,3]. The game of hurling has been described as an intermittent field game similar to other team sports [3,4,5,6,7]. Even though the game is hundreds of years old, research has only recently described the match-play performances of competition [1,8,9,10] and training [11,12,13]. Using Global Positioning Systems (GPS) technology, the match-play running performances were quantified in youth, sub-elite and elite hurlers [1,2,8,14,15,16]. Typically, the total distance, high-speed running (HSR), sprint distance and the number of accelerations and decelerations have been used to describe the running performances in hurling [1,2,8,14,15,16]. Furthermore, a parameter called dynamic stress load, which summarizes the accumulation of the rates of players’ accelerations has also been used within team sport [17]. Measuring the mechanical stress (e.g., dynamic stress load) of the player has shown a better correlation with internal training load variables compared to total distance, metabolic power and high-intensity running [18]. Knowledge of the competition-demands can be used to design hurling-specific conditioning programmes so that players can be optimally prepared [19].

Due to COVID-19 restrictions, the rules about the match-structure of hurling have changed. Hurling games are now played over four quarters instead of two halves [16]. The research presented in hurling so far focuses on the match demands over the full game and per half [1,2,8,9,20]. With the change in the game structure, the current literature fails to inform coaches about the per-quarter running demands of hurling, therefore knowledge of the between-quarter match demands is limited. Only one study has recently attempted to profile the game of hurling per quarter [16]. Results showed that compared to Q1, total distance was lower in Q3, and high-speed running reduced in all quarters (Q2–Q4) compared to Q1 [16]. However, the previously used parameters present distance within set speed thresholds, which fails to describe the number of times the players actually change speed. In hurling, players are required to contest for possession against their direct opponent, which usually consists of numerous starting and stopping to gain possession. Therefore, quantifying accelerations, decelerations and dynamic stress load (i.e., the weighted accelerometery load above 2G) will inform coaches about the changes in velocity for each player. Furthermore, these per-quarter data would provide coaches with necessary information about the fluctuations in velocity during the game and help identify the most intense periods of the game [15].

**Figure 1:**
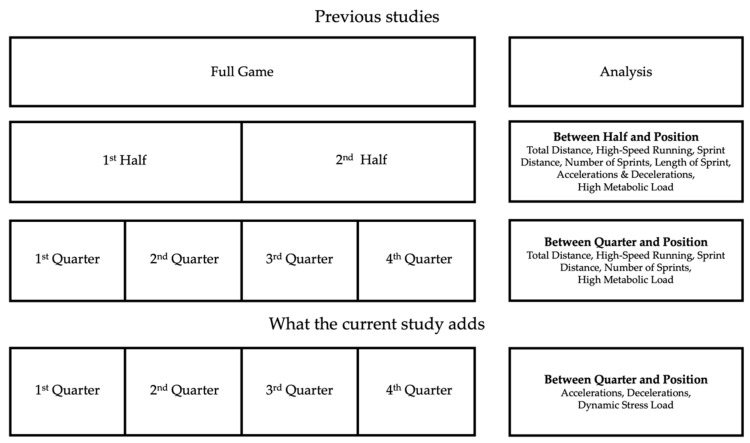
Graphical representation of what the current study adds to the literature.

The between-position differences for the full game and per half were previously reported in hurling at U17 [20], U21 [2] and senior [1,8,9]. The per quarter match-demands in senior hurling showed that all positions experienced a temporal decrement in all metrics in at least one quarter except midfielders’ total distance and full-forwards’ high-speed running [16]. However, no data exists for the number of accelerations and decelerations and dynamic stress load between quarters within positions. Therefore, the aims of the current study were to describe the differences in the number of accelerations and decelerations and dynamic stress load between quarters of match-play and within playing positions in elite senior hurling. A graphical representation of what the current study will add to the literature is displayed in Figure 1. It was hypothesized that the number of accelerations and decelerations and dynamic stress load would decrease as the match progresses. Furthermore, it was hypothesized that there would be a temporal decrement within position between quarters. 

## 2. Materials and Methods

### 2.1. Participants and Design

Forty-two (*n* = 42) elite male hurlers (age; 27 ± 5 years, height; 182 ± 6 cm, and body mass; 87 ± 6) volunteered to participate in the present study. All players in the current study were members of the county’s squad that season (2018–2020). Players were chosen from the elite squad as this is the highest possible level that players can compete at. All participants had completed at least a 6-week preseason conditioning program (gym and pitch sessions). Those players on the squad who were injured were not included in the study. Data were only included if a full match (70-minute; 4 quarters) was completed. The elite hurlers were divided into five playing positions: full backs, half backs, midfielders, half forwards and full forwards. All games (*n* = 22) took place between 14.00 and 21.00 hours. The specific running performance variables were collected with GPS. After ethical approval, the purpose, procedures and potential risks involved in the study were explained. The subjects were informed that they could withdraw from the study at any time. A medical declaration and written informed consent were completed in line with the procedures set by the Limerick Institute of Technology Research Ethics Committee. All procedures were approved by the local Ethics Committee, and the study was conducted according to the Declaration of Helsinki (1975) for studies involving human subjects.

### 2.2. Procedures

During the familiarization session, the subjects height and body mass was measured using a stadiometer (Seca 217, Seca Ltd., Hamburg, Germany) and Seca Weighing Scales (Seca Ltd., Hamburg, Germany), respectively. A 10 Hz GPS unit and 100 Hz triaxial accelerometer (STATSports, Northern Ireland, Apex) was used to collect the running performances [2,15,16]. The number of satellites and the horizontal dilution of precision was 19 ± 7 and 1.3 ± 0.2, respectively, across all games. GPS data were downloaded and further analyzed using the STATSports, Apex software (Firmware 2.5). The GPS units’ validity and reliability have been previously established [21,22]. The GPS unit (dimensions: 86 mm × 33 mm × 14 mm, mass 50 g) was placed in a sports vest and worn under the playing jersey [1]. The GPS were activated, and a satellite lock were established 15 min before the warm-up [23]. Prior to data collection, the participants were familiarized with the GPS technology [1,16].

GPS units were used to collect the number of accelerations (>3 m·s^−2^) and decelerations (>3 m·s^−2^) [19] and the dynamic stress load expressed in arbitrary units (AU). Dynamic stress load is recorded by a 100 Hz triaxial accelerometer measuring accelerations in the three movement axes (X, Y and Z planes) [24]. The dynamic-stress load metric was calculated automatically using a custom algorithm included in the STATSports GPS software (STATSport, Northern Ireland, Apex Software 2.1.15). Following each match the GPS data were downloaded using the STATSport analysis software [1]. Once the GPS data were download the unit name was labelled as the playing position. Quarter 1, 2, 3 and 4 data were identified by a timestamp and manually exported into a Microsoft Excel spreadsheet (Microsoft, Redmond, WA, USA).

### 2.3. Statistical Analysis

Statistical analysis was performed using a statistical software package (SPSS for Windows, Version 22, SPSS Inc., Chicago, IL, USA). Descriptive analysis and assumptions of normality were verified prior to parametric statistical analysis. The analysis was performed using a two-way (quarter × position) mixed-model design. A Bonferroni post-hoc correction was used to detect differences between positions (five levels: full backs, half backs, midfielders, half forwards, full forwards) and match quarters when an interaction occurred [16]. The dependent variables included the number of accelerations and decelerations, and dynamic stress load. The independent variables were match quarters and playing positions. Standardized effect sizes (ES) were calculated as trivial (≤0.20), small (0.21–0.60), moderate (0.61–1.20), large (1.21–2.00) and very large (2.01–4.00) [25]. Statistical significance was set at an accepted level of α < 0.05.

## 3. Results

**Figure 2:**
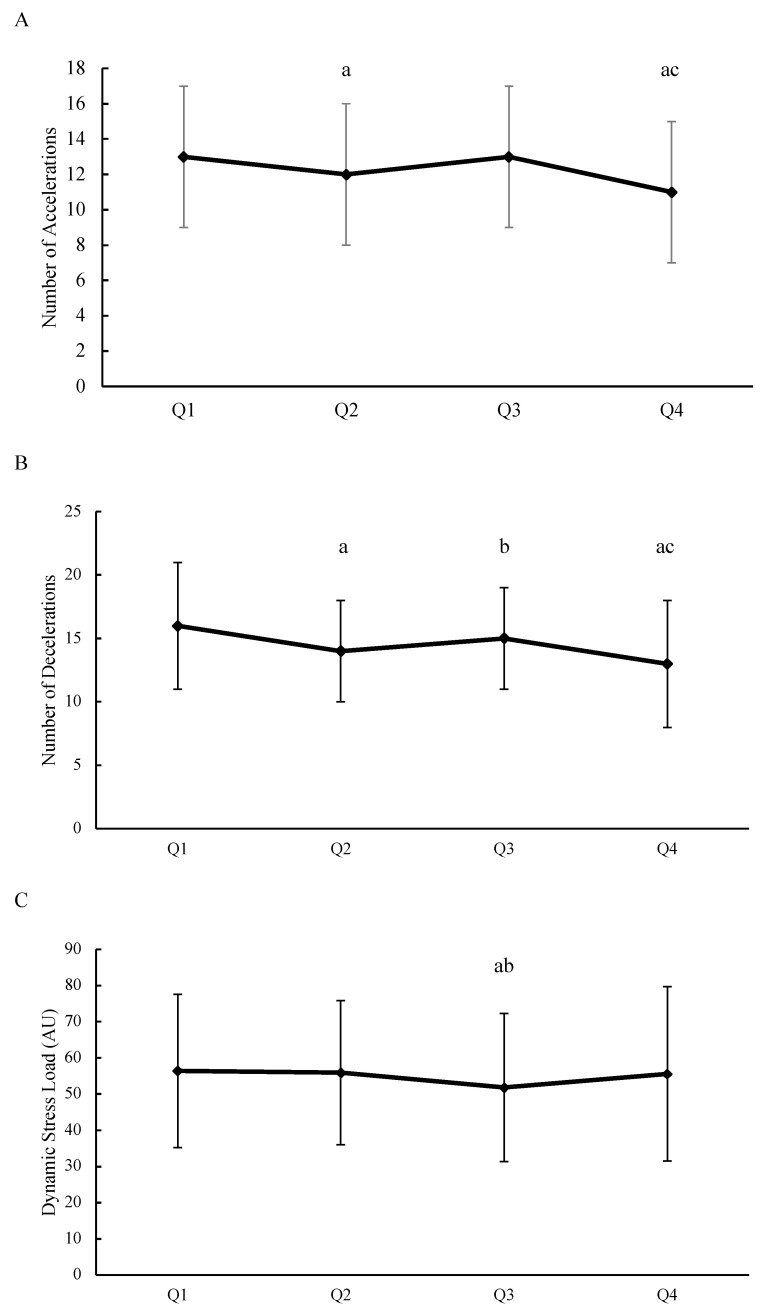
Mean ± SD of (**A**) the number of accelerations, (**B**) decelerations and (**C**) the dynamic stress load per quarter. Q = quarter; AU = Arbitrary units; a = different from quarter 1; b = different from quarter 2; c = different from quarter 3.

During the full game, players performed 49 ± 11 accelerations and 58 ± 14 decelerations, with a dynamic stress load of 220 ± 78 (AU). The number of accelerations and decelerations and dynamic stress load per quarter are presented in Figure 2.

The number of accelerations was 13 ± 4 in Q1, 12 ± 4 in Q2, 13 ± 4 in Q3 and 11 ± 4 in Q4. A higher number of accelerations were performed in Q1 compared to Q2 (ES = 0.28), and Q4 (ES = 0.57) and in Q3 compared to Q4 (ES = 0.50). The number of decelerations was 16 ± 5 in Q1, 14 ± 4 in Q2, 15 ± 4 in Q3 and 13 ± 5 in Q4. A higher number of decelerations were performed in Q1 compared to Q2 (ES = 0.44), and Q4 (ES = 0.60) and in Q3 compared to Q4 (ES = 0.44). The dynamic stress load was 56 ± 21 AU in Q1, 56 ± 20 AU in Q2, 52 ± 20 AU in Q3 and 56 ± 24 AU in Q4. There was a decrease in dynamic stress load in Q3 compared to Q1 (ES = −0.20) and Q2 (ES = −0.20).

**Figure 3:**
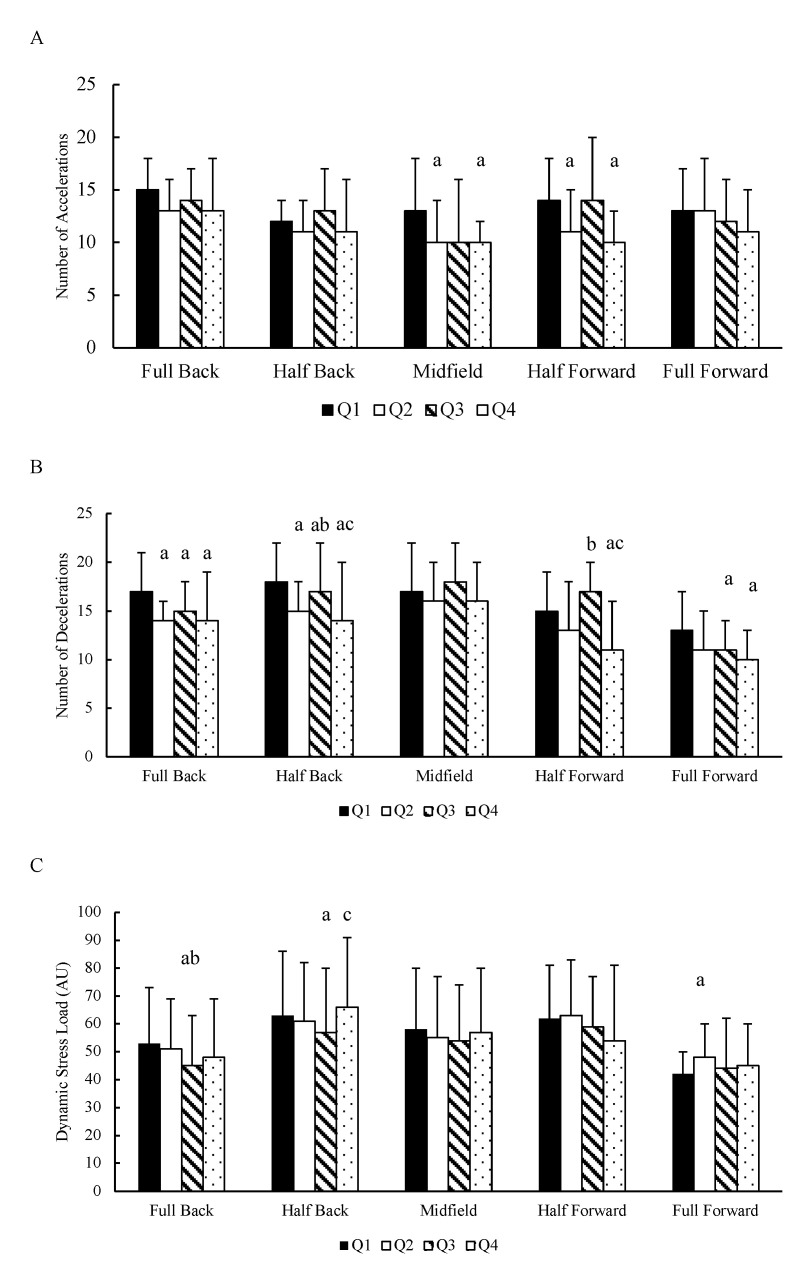
Mean ± SD of the number of (**A**) accelerations, (**B**) decelerations and (**C**) the dynamic stress load per position per quarter. Q = quarter; AU = Arbitrary units; a = different from quarter 1; b = different from quarter 2; c = different from quarter 3.

The differences between quarters for each playing position are presented in Figure 3. A quarter × position interaction was found for the number of accelerations (*p* = 0.001). A main effect was found for quarter (*p* < 0.001), and position (*p* = 0.005). Midfielders (ES = 0.66 and ES = 0.79, respectively) and half forwards (ES = 0.75 and ES = 1.13, respectively) performed a greater number of accelerations in Q1 compared to Q2 and Q4. There were no other differences (*p* > 0.05) between quarters in the number of accelerations. Full backs (ES = 0.95), and half backs (ES = 0.85) performed a greater number of decelerations in Q1 compared to Q2.

A quarter × position interaction was found for the number of decelerations (*p* < 0.001). A main effect was found for quarter (*p* < 0.001), and position (*p* < 0.001). Full backs (ES = 0.57), and full forwards (ES = 0.57) performed a greater number of decelerations in Q1 compared to Q3. Full backs (ES = 0.66), half backs (ES = 0.78), half forwards (ES = 0.88) and full forwards (ES = 0.85) performed a lower number of decelerations in Q4 compared to Q1. Half backs and half forwards performed a greater number of decelerations in Q3 compared to Q2 (ES = 0.49 and ES = 0.97, respectively) and Q4 (ES = 0.54 and ES = 1.46, respectively). There were no other differences (*p* > 0.05) in the number of decelerations between quarters in any position.

A quarter × position interaction was found for dynamic stress load (*p* = 0.004). A main effect was found for quarter (*p* = 0.001), and position (*p* = 0.003). The dynamic stress load was lower in Q3 compared to Q1 (ES = −0.42) and Q2 (ES = −0.33) full backs and Q1 (ES = −0.26) and Q4 (ES = 0.37) in half backs. Full forward’s dynamic stress load was higher in Q2 compare to Q1 (ES = 0.59).

## 4. Discussion

Hurling games are now played over four quarters instead of two halves due to COVID-19 restrictions [16]. The current study aimed to describe the differences in the number of accelerations and decelerations and dynamic stress load between quarters of match-play and within playing positions in elite senior hurling. As hypothesized, there was a decrement in the running performance as the match progressed. Specifically, the main findings showed that there was a decrement in the number of accelerations and decelerations in Q2 and Q4 compared to Q1 and Q3 respectively. The only decrement in dynamic stress load occurred in Q3. All positions experienced some temporal decrement in the number of accelerations and decelerations and dynamic stress load between at least one quarter. The current findings will provide coaches with new information about the decrement in running performance across elite level hurling match-play in accordance with the present regulations.

The results showed a temporal decrement in the number of accelerations and decelerations in Q2 and Q4 compared to Q1 and Q3. Previous research in hurling also found greater high-speed running and high-metabolic load distance in Q1 compared to other quarters (Q2–Q4) [16]. The half-time period of 15 minutes seems to provide hurlers with enough time to recover from the first two quarters, as players were able to perform a similar number of accelerations and decelerations in Q3 compared to Q1 [16]. In the final quarter, the number of accelerations and decelerations were lower than Q1 and Q3. Similar results were found in Australian Football, where the number of accelerations and decelerations were shown to reduce towards the end of the game [26]. In the final quarter, the players would have completed at least fifty-five minutes of high-speed running, sprint distance, and maximum accelerations and decelerations in the previous three quarters. With this accumulation of high-intensity distance it is likely that the players are experiencing some fatigue in the last quarter. The drop-offs in the current study are similar to reported in soccer, where it was suggested that fatigue occurred temporarily throughout and towards the end of a game [27]. The current results provide coaches with the knowledge of the acceleration and deceleration demands across each quarter in hurling. Previous research has described hurling as a start–stop game, with the ball going out-of-play frequently (every ~31 s) and the players changing speeds [14]. Coaches could use small-sided games [28] of short durations, where players are accelerating frequently to replicate the number of accelerations and decelerations that occur in the match. Furthermore, coaches could prescribe activities where players perform fast accelerations and decelerations towards the end of training following the volume of high-intensity distance accumulated earlier in the session so that players become accustomed to performing fast changes in speed towards the end of games.

It has been suggested that dynamic stress load, a measure of the mechanical stress on the body recorded from the number of impacts and forces applied through ground contacts, can be used to measure external load in team sport athletes [27]. For the first time, the dynamic stress load was recorded during competitive hurling matches so a direct comparison cannot be made. The dynamic stress load for the full game was lower than that found in soccer (~343 AU) [27]. The overall match duration in hurling is twenty minutes shorter compared to soccer, which may explain the difference between sports. Similar to number of accelerations and decelerations, between-quarter differences were observed in dynamic stress load. Even though the number of accelerations were higher in Q3, the dynamic stress load was lower in Q3 compared to Q1 and Q2. Previous research in hurling has shown that players performed a lower high-speed- and sprint-distance in Q3, which may explain the reduction in dynamic stress load in Q3. In addition, to the author’s knowledge no study has compared the dynamic stress load between quarters in other sports. During matches which include physical contact like hurling, many players do not like to wear heart-rate monitors. As a result, the monitoring of internal workload can be difficult to collect. Previous research has suggested that dynamic stress load can be an effective metric to monitor fatigue in team sports [27]. Hurling coaches could use the current results to design training activities to help players minimize the decrement in the final stages of the match.

There were between-quarter differences observed for the number of accelerations and decelerations and dynamic stress load within positions. Midfielders and half forwards performed more accelerations in Q1 compared to Q2 and Q4, whereas full backs and half backs performed more decelerations in Q1 compared to Q2, Q3 and Q4. In addition, the number of deceleratons in Q1 within full forwards was also more demanding compared to Q3 and Q4. Less differences between quarters was observed for dynamic stress load within positions, yet Q1 was the highest in full backs, half backs and full forwards. No other research is available which described the number of accelerations (>3 m·s^−2^) and decelerations (>3 m·s^−2^) and dynamic stress load between positions across quarters in hurling, making direct comparisions difficult. However, previous research has found similar trends between quarters in hurling, where the high-speed running- and high metabolic load-distance were greater in Q1 compared to Q2, Q3 and Q4 across most positions [16]. Hurlers are at their freshest at the start of the game, which may allow them to cover greater distances compared to other quarters. The current results for dynamic stress load can help coaches identify the positions which are most demanding. Once an individual dynamic stress load profile is developed for each player, coaches can target additional conditioning for players in these positions. In addition, as the current data are presented for each quarter, a substitution policy could be developed for positions that are inclined to experience the most decrement.

The present study comes with some acknowledged limitations. Firstly, the duration of when the ball was in-play was not recorded in this study. When the ball is out-of-play, this limits the opportunities that players have to accelerate and decelerate. Differences in the ball-in-play time between quarters may have affected the results. Future studies should quantify the number of accelerations and decelerations per min of ball-in-play and ball-out-of-play between quarters. Secondly, no attempt was made to account for the technical and tactical differences between quarters. Knowledge of when and where the accelerations and decelerations occur in relations to the ball would be interesting so that hurling-specific activities could be developed. Finally, the dynamic stress load metric measures the force and impact when the feet contact the ground. As players tire, their foot contacts may be longer on the ground, causing an increase in dynamic stress load. Therefore, although this metric is valuable in providing an overview of the stress placed on the player during a game, it can vary between players depending on their fitness level. It must be noted that comparing the dynamic stress load between positions can be challenging. Due to the potential variability between players’ fitness levels, the between-position analysis of the dynamic stress load metric was not included in the present study.

Some important practical applications appear considering the current findings. The results show that players are required to accelerate and decelerate throughout all four quarters of the game. Therefore, the conditioning of hurlers needs to include activities that challenge the players to increase and decrease their speed frequently. Coaches should consider the exercise selection that take place towards the third and fourth quarters of training. Players may not be required to perform maximal accelerations and decelerations during certain activities especially towards the end of training. This may leave them underprepared for the maximal changes in speed that occur during a game. Including activities where the players have to compete against each other may help to motivate them to change speed quickly. Conditioning for midfielders and half forwards should ensure that they can repeat the number of accelerations in each quarter. Coaches should consider the distances of activities that they set-up so that the players can perform maximum accelerations especially in hurling drills. The distance of the activity may be too short, or the ball might be approaching the player to quickly, this may limit the opportunity for him to maximally accelerate and therefore decelerate.

## 5. Conclusions

The current study was the first to provide an insight into the temporal decrements in the number of accelerations and decelerations and dynamic stress load between quarters in elite hurling. The findings showed that the number of accelerations and decelerations decreased in Q2 and Q4 compared to Q1 and Q3. The dynamic stress load experienced a decrement in Q3. Full backs, half backs and full forwards performed a similar number of accelerations in each quarter, whereas only midfielders performed a similar number of decelerations in each quarter. The results of this study provide coaches with new information about the fluctuations in running intensity that occurs during the game. This knowledge may be used to help in the design of specific conditioning programs for elite hurlers.

## Data Availability

The data presented in this study are available on request from the corresponding author.
